# Natural-Fiber-Reinforced Chitosan, Chitosan Blends and Their Nanocomposites for Various Advanced Applications

**DOI:** 10.3390/polym14050874

**Published:** 2022-02-23

**Authors:** Rushdan Ahmad Ilyas, Humaira Alias Aisyah, Abu Hassan Nordin, Norzita Ngadi, Mohamed Yusoff Mohd Zuhri, Muhammad Rizal Muhammad Asyraf, Salit Mohd Sapuan, Edi Syams Zainudin, Shubham Sharma, Hairul Abral, Mochamad Asrofi, Edi Syafri, Nasmi Herlina Sari, Mazlan Rafidah, Sharifah Zarina Syed Zakaria, Muhammad Rizal Razman, Nuriah Abd Majid, Zuliskandar Ramli, Ashraf Azmi, Sneh Punia Bangar, Rushdan Ibrahim

**Affiliations:** 1 Faculty of Engineering, School of Chemical and Energy Engineering, Universiti Teknologi Malaysia (UTM), Johor Bahru 81310, Johor, Malaysia; abuhassannordin@gmail.com (A.H.N.); norzita@cheme.utm.my (N.N.); 2 Centre for Advanced Composite Materials (CACM), Universiti Teknologi Malaysia (UTM), Johor Bahru 81310, Johor, Malaysia; 3 Institute of Tropical Forestry and Forest Products, Universiti Putra Malaysia (UPM), Serdang 43400, Selangor, Malaysia; sapuan@upm.edu.my (S.M.S.); edisyam@upm.edu.my (E.S.Z.); 4 Advanced Engineering Materials and Composites Research Centre (AEMC), Department of Mechanical and Manufacturing Engineering, Universiti Putra Malaysia (UPM), Serdang 43400, Selangor, Malaysia; 5 Institute of Energy Infrastructure (IEI), Universiti Tenaga Nasional, Jalan IKRAM-UNITEN, Kajang 43000, Selangor, Malaysia; asyrafriz96@gmail.com; 6 Department of Mechanical Engineering, IK Gujral Punjab Technical University, Kapurthala 144603, India; shubham543sharma@gmail.com; 7 Department of Mechanical Engineering, Andalas University, Padang 25163, Sumatera Barat, Indonesia; habral@yahoo.com; 8 Department of Mechanical Engineering, University of Jember, Kampus Tegalboto, Jember 68121, East Java, Indonesia; asrofi.teknik@unej.ac.id; 9 Department of Agricultural Technology, Agricultural Polytechnic, Payakumbuh 26271, West Sumatra, Indonesia; edisyafri11@gmail.com; 10 Mechanical Engineering Department, Faculty of Engineering, University of Mataram, Mataram 83115, West Nusa Tenggara, Indonesia; n.herlinasari@unram.ac.id; 11 Department of Civil Engineering, Universiti Putra Malaysia (UPM), Serdang 43400, Selangor, Malaysia; rafidahmazlan16@gmail.com; 12 Research Centre for Environment, Economic and Social Sustainability (KASES), Institute for Environment and Development (LESTARI), Universiti Kebangsaan Malaysia (UKM), Bangi 43600, Selangor, Malaysia; szarina@ukm.edu.my (S.Z.S.Z.); nuriah@ukm.edu.my (N.A.M.); 13 Research Centre for Sustainability Science and Governance (SGK), Institute for Environment and Development (LESTARI), Universiti Kebangsaan Malaysia (UKM), Bangi 43600, Selangor, Malaysia; mrizal@ukm.edu.my; 14 Institute of the Malay World and Civilisation (ATMA), Universiti Kebangsaan Malaysia (UKM), Bangi 43600, Selangor, Malaysia; ziskandar@ukm.edu.my; 15 School of Chemical Engineering, College of Engineering, Universiti Teknologi MARA, Shah Alam 40450, Selangor, Malaysia; ashraf.azmi@uitm.edu.my; 16 Department of Food, Nutrition and Packaging Sciences, Clemson University, Clemson, SC 29631, USA; snehpunia69@gmail.com; 17 Pulp and Paper Branch, Forest Research Institute Malaysia, Kepong 52109, Selangor, Malaysia; rushdan@frim.gov.my

**Keywords:** natural fiber, chitosan, chitosan blends, chitosan nanocomposites, cellulose, nanocellulose

## Abstract

There has been much effort to provide eco-friendly and biodegradable materials for the next generation of composite products owing to global environmental concerns and increased awareness of renewable green resources. This review article uniquely highlights the use of green composites from natural fiber, particularly with regard to the development and characterization of chitosan, natural-fiber-reinforced chitosan biopolymer, chitosan blends, and chitosan nanocomposites. Natural fiber composites have a number of advantages such as durability, low cost, low weight, high specific strength, non-abrasiveness, equitably good mechanical properties, environmental friendliness, and biodegradability. Findings revealed that chitosan is a natural fiber that falls to the animal fiber category. As it has a biomaterial form, chitosan can be presented as hydrogels, sponges, film, and porous membrane. There are different processing methods in the preparation of chitosan composites such as solution and solvent casting, dipping and spray coating, freeze casting and drying, layer-by-layer preparation, and extrusion. It was also reported that the developed chitosan-based composites possess high thermal stability, as well as good chemical and physical properties. In these regards, chitosan-based “green” composites have wide applicability and potential in the industry of biomedicine, cosmetology, papermaking, wastewater treatment, agriculture, and pharmaceuticals.

## 1. Introduction

Nowadays, ecological concerns have resulted in renewed interest in natural materials. Recyclability and environmental safety are becoming increasingly important in the consideration of a better future in sustainability [1,2,3,4]. Hence, the need for more versatile polymer-based materials has led to increasing interest in polymer composites filled with natural, organic fillers, for example, fillers that are biodegradable and come from renewable sources [5,6,7,8]. Day by day, biomaterials are crucial in the development of a sustainable environment. Even though biomaterials are newly in development for the delivery of drugs, tissue engineering, and medical diagnostics, but there has been good improvement for both physical and chemical methods that can manage biological responses [9].

The most suitable definition for a biomaterial is any substance that is not a drug or synthetic substance that can be used any time to partially or totally replace any tissue or other parts of body, which can improve the quality of life for an individual [10]. There are four different types of biomaterials that are commonly known in industries, which are polymers, metals, ceramics, and composites.

In general, chitosan is a sugar that is contained in the hard outer skeleton of shellfish such as crab, lobster, and shrimp, which is used in medication [11,12]. In fact, chitosan is a derivative from chitin, and it is one of the most bountiful natural biopolymers as opposed to cellulose [10]. The composition of chitin in seashell waste consists of proteins (30–40%) calcium carbonate and calcium phosphate (30–50%), and chitin (20–30%) [10,13]. This review paper discusses green composites of natural fibers in detail and also discusses the development and characterization of chitosan, natural-fiber-reinforced chitosan biopolymers, chitosan blends, as well as chitosan nanocomposites.

## 2. Natural Fiber

Natural fiber is a lignocellulosic material which is mainly composed of cellulose, hemicelluloses, lignin, pectin, wax, ash, and moisture [14,15,16]. It is important to understand the composition because the mechanical properties of natural fibers are dependent on it, as stated by Farok et al. [17]. From previous study, Sinha et al. [18] stated that natural fibers are hair-like or thread-like naturally existing substances with a high aspect ratio, and the application of these fibers is in high demand due to their advantages such as low cost, low weight, and biodegradability. However, natural fibers also come with drawbacks, which includes their hydrophilic nature [19,20,21,22]. The hydroxyl group will absorb the moisture and prevent from damage and degradation [23,24,25,26,27,28].

According to Sinha et al. [18], natural fiber is classified into three categories, which are animal, vegetable, and mineral fibers. Natural fibers such as abaca, cotton, jute, flax, hemp, and coir are deployed for different industrial applications. Recently, there has been in rapid growth in research and innovation in natural fiber composites in multidisciplinary areas [29,30,31,32,33]. The characteristics of natural fiber composites are durability, low cost, low weight, high specific strength, non-abrasiveness, equitably good mechanical properties, eco-friendliness, and biodegradability [6,29,34,35]. These materials possess promising potential for a wide range of industries, including medical, structural and construction, packaging, military, aerospace, and automobile industries [36,37,38,39,40,41,42,43,44,45,46,47].

### 2.1. Types of Green Composites and Chemical Composition of Natural Fibers

Green composites are a type of biocomposites in which natural fibers are used to strengthen a bio-based polymer matrix [48]. Animal fiber is extracted from the fur of animals, mineral fiber is a naturally occurring fiber or modified fiber produced from minerals, while the main content of plant fiber is cellulose [49,50]. Examples of these classifications are shown in Table 1.

Natural fiber manufacturing is expanding worldwide as the product base expands. Table 2 lists the major manufacturers as well as the yearly outputs of these fibers across the globe [51]. Chemical composition refers to the arrangement of particles and the types and ratios of atoms in chemical substances where the composition is different when there is an addition or subtraction of chemicals, and when the ratios changes. Table 3 shows the chemical composition of natural fibers. The common chemical constituents in green or natural fibers are cellulose, hemicelluloses, lignin, pectin, and wax. All natural fibers have similar components but different compositions, which make them behave differently [52,53,54,55].

According to Table 3, different types of fiber consist of different compositions of cellulose, hemicellulose, lignin, pectin, wax, ash, moisture, and other contents. For cellulose, bamboo [57] contains the highest composition, which is 78.83%, followed by sisal, which is 65.8%, and the lowest one is coconut, which only contains 37–43% cellulose. For hemicellulose, coconut and abaca are the highest, the percentages of which are around 24% to 29%. Coconut also has the highest content of lignin, up until 28%, and is followed by kenaf, which is 20.1%, and the lowest is jute, which is only 0,2%. Meanwhile for pectin, wax, ash, moisture, and others, there are only a few fibers that contain these compositions, such as jute, sisal, kenaf, and coconut [58,59].

### 2.2. Mechanical Properties of Green Fibers

Generally, mechanical properties give meaning to the physical properties which the materials show as forces are applied; examples of these properties are elasticity, strength of tensile, elongation, hardness, and fatigue limit. Nowadays, natural or green fibers are widely used for production in a few types of applications, including automotive, aircraft, construction, and building [29,60,61,62,63]. Thus, mechanical properties of natural fibers are crucial so that benefits can be utilized as much as possible. As well as advantages, natural fibers also have major drawbacks such as their hydrophilic nature, which means they have a high degree of moisture absorption and poor dimensional stability. Few studies have comprehensively discussed the limitations of natural fiber composites such as compatibility with polymers, low thermal properties, as well as irregular properties [64,65,66]. Table 4 shows the mechanical and physical properties of natural fibers.

The physical and mechanical properties of natural fibers consist of the diameter of fiber, density of the fibers, tensile strength, and the Young’s modulus value of the fiber. For diameter [67], jute fiber has the greatest diameter, which is around 250 μm to 2500 μm. This is followed by coconut. which has a value of 396.98 μm, and kenaf has the lowest value, which is 83.5 μm. Abaca, bamboo and jute fibers have the highest density, which is around 1.5 g/cm^3^. For the tensile strength which represents the resistance of fibers, jute’s tensile strength can reach 800 MPa. This is followed by abaca and bamboo, with values of 717 MPa and 500–575 MPa, respectively [68]. The Young’s Modulus represents the elasticity of fiber, and bamboo has the highest value of Young’s Modulus, with the value of 27–40 GPa [69]. Since the tensile strength is higher, it used as reinforcement in order to improve or upgrade the mechanical properties of a composite such as cement mortar and polymer-reinforced composites [70,71,72]. Abaca fiber also has higher tensile strength, which is about 717 MPa, and its strength is good for any natural fibers. Thus, it is usually used in the production of the exterior of passenger vehicles, where it can resist stone strikes [73,74] However, abaca fibers have not been fully explored to their fullest potential as a composite [18].

## 3. Chitosan

### 3.1. Advantages and Disadvantages of Chitosan

As a biomaterial, chitosan provides many advantages and disadvantages. As a biomaterial form, chitosan can be made into a few forms, such as hydrogels, sponges, films which appear in 3D forms, and also porous membrane which appears in 2D form, and each of them have specific applications in industries [75,76]. The advantages and disadvantages for each of the type are shown in Table 5.

### 3.2. Chemical and Physical properties of Chitosan

**Figure 1:**
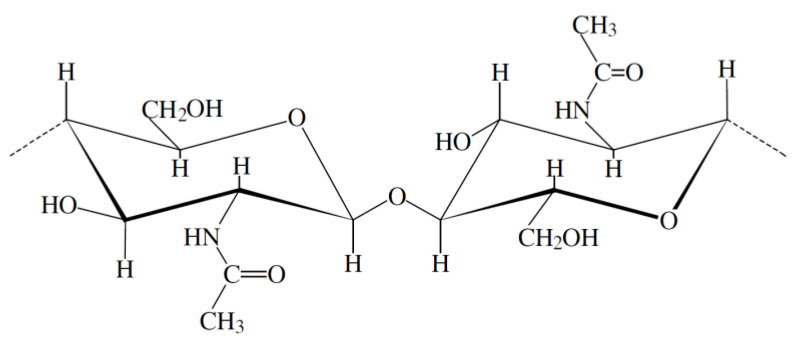
Structure of chitin.

**Figure 2:**
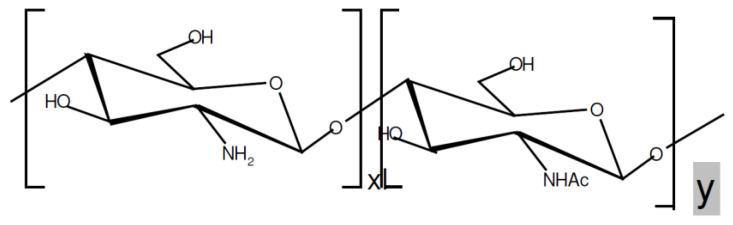
Partially deacetylated chitin.

**Figure 3:**
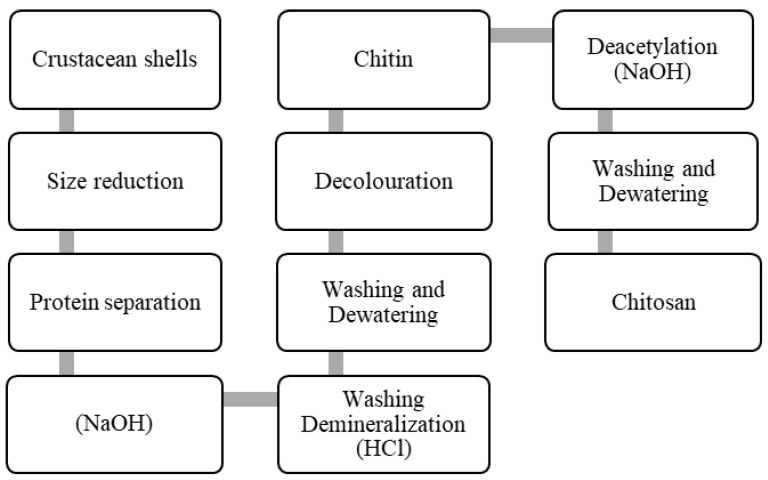
Making of chitosan.

After cellulose, chitin is the second most ubiquitous natural polysaccharide on Earth and is composed of β(1→4)-linked 2-acetamido-2-deoxy-β-D-glucose1 (N-acetylglucosamine) (Figure 1). It is often considered as a cellulose derivative, even though it does not occur in organisms producing cellulose. It is structurally identical to cellulose, but it has acetamide groups (−NHCOCH_3_) at the C-2 positions. Similarly, the principle derivative of chitin, chitosan, is a linear polymer of α (1→4)-linked 2-amino-2-deoxy-β-D-glucopyranose and is easily derived by N-deacetylation, to a varying extent that is characterized by the degree of deacetylation, and is consequently a copolymer of N-acetylglucosamine and glucosamine (Figure 2). Chitin is estimated to be produced annually almost as much as cellulose [77]. It has become of great interest not only as an under-utilized resource but also as a new functional biomaterial of high potential in various fields, and the recent progress in chitin chemistry is quite significant. The production of chitosan from crustacean shells obtained as a food industry waste is economically feasible, especially if it includes the recovery of carotenoids. Figure 3 displays the process of making chitosan from crustacean shells.

Generally, chitosan is a derivative from chitin, where a certain group of polymers deacetylated from chitin. Hence, chitin and chitosan are different based on the degree of deacetylation [78,79]. In addition, the production of chitosan is mainly based on a further reaction of chitin. Besides, the properties of every chitosan produced is different due to different raw materials [80,81]. However, the qualities can be measured using same properties such as viscosity, deacetylation, molecular weight, and polymorphous structure [82]. The physical properties of chitosan-based polymer are shown in Table 6.

### 3.3. Mechanical Properties of Chitosan

To comprehend the mechanical behavior of chitosan-based films, the mechanical properties of chitosan films must be investigated. The efficiency and integrity of the films are determined by their tensile strength and percentage elongation at break during preparation, use, and handling. Since chitin and chitosan have poor mechanical strength, it is necessary to change some of their properties so that they can be used as bioadhesives, nanocomposites, and waste materials that pollute the atmosphere [86]. Based on Table 7, the highest value of tensile strength is for chitosan—spirulina extract (21.24 MPa–29.65MPa) with percentage range between 2.5% and 50.0%. However, the higher elongation is for chitosan—graphene oxide, with 57.34% to 72.70% and less than 2%.

### 3.4. Thermal Properties of Chitosan

**Figure 4:**
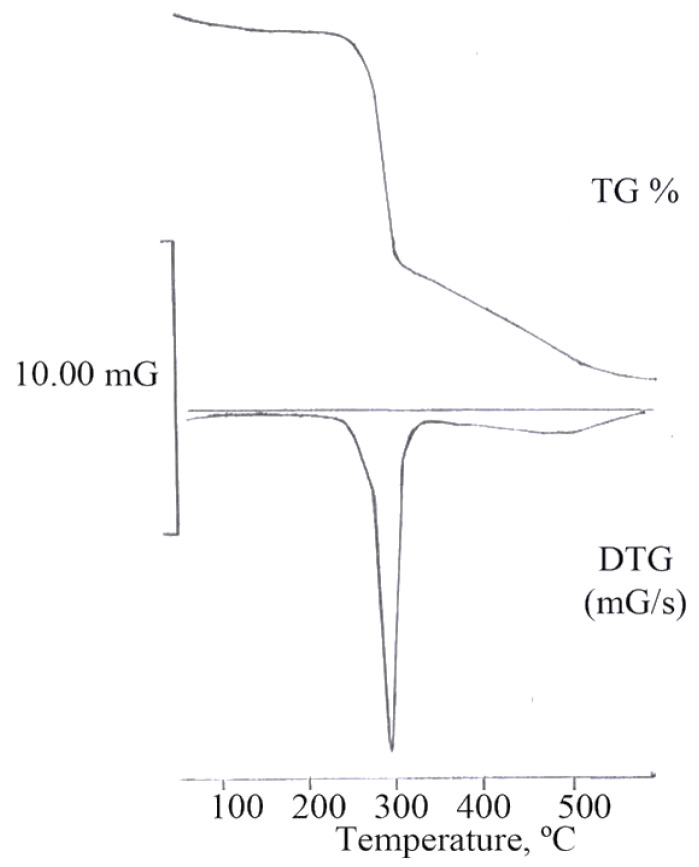
Thermogravimetric curves for chitosan [95]. (Mostafa Amin, 2012).

Chitosan exhibits a high sensitivity to numerous types of degradation, including thermodegradation. Thermal study revealed that this biopolymer cannot resist temperatures beyond 200–220 °C [91,92]. Figure 4 depicts the thermogravimetry (TG) curve of chitosan. In a chitosan polymer, there are two levels of degradation. Weight reduction in the first stage begins at 220 °C and progresses to 320 °C, with a 50 percent weight loss. The overall weight loss rate is measured by derivative equipment. At 295 °C, there is a reaction connected with the TG apparatus. The second stage achieves a mean temperature of 470 °C, with a weight loss of 40%. The activation energy of the degradation of chitosan was found to be 52.2 kJ/mol [93,94].

## 4. Processing of Chitosan Green Composites

As chitosan is created from the derivation of chitin, the usual industrial process that are applied for the extraction of chitin involve three main steps; the deproteinization of raw material with the addition of alkaline solution, followed by demineralization through a treatment using acidic solution, and lastly the discoloration of product obtained through the treatment using alkaline solution [96]. There are different ways of converting chitin to chitosan, such as through an enzymatic method or a process of chemical conversion, but between these two methods, the chemical process is more preferable since the cost is a lot cheaper compared to the other method and also when considering the production capacity of chitosan [96,97].

According to Muxika et al. [96], chitosan is a substance or copolymer that comes from deacetylation using alkaline from chitin, where it formed by _D_-glucosamine and N-acetyl-_D_-glucosamine units, which are also linked by ß-1, 4 glycosidic linkages, and the solubility of chitosan allows it to be produced in various forms such as films, nanofibers, hydrogels, or pastes. In the biomaterial industry, chitosan production provides many applications based on the structure and forms [98,99]. There are various types of chitosan processing methods, such as solution casting, dipping and spray coating, compression molding, freeze casting and drying, blending, layer-by-layer processing, and also, rapid prototyping. A few of the techniques or processes are further discussed.

### 4.1. Solution and Solvent Casting

**Figure 5:**
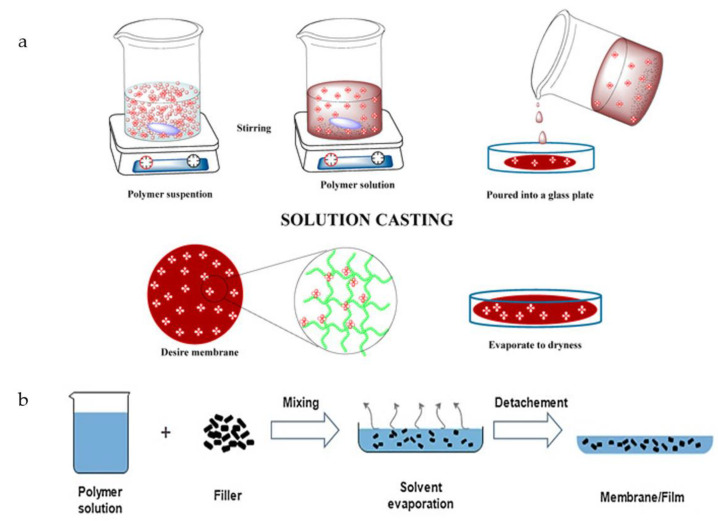
General steps in the (**a**) solution casting and (**b**) solvent casting method for composite fabrication [102,103].

The solution casting method is the most commonly known method for chitosan processing. This process involves an acidic solution in which the chitosan powder is dissolved and poured into a Petri dish (Figure 5). This process needs to be carried out in dry conditions at room temperature or in an oven at a certain temperature until the film is completely dry and peels off the mold by itself [96]. Through the application of multisolution coatings on glass substrate, this technique is not only appropriate for the production of single-layered film or membrane, but also for the fabrication of multilayered, dense films. The addition of highly volatile solvents to the casting solution followed by an evaporation stage before phase inversion in a non-solvent immersion may aid in the development of a top dense layer. However, even though this method is commonly used as a method or technique to prepare chitosan films at a small scale, further research must be done to analyze the possibility for the manufacturing scale production of chitosan [100]. For the production of different kinds of nanocomposite membranes, the solution casting approach has been widely researched [101].

### 4.2. Dipping and Spray Coating

**Figure 6:**
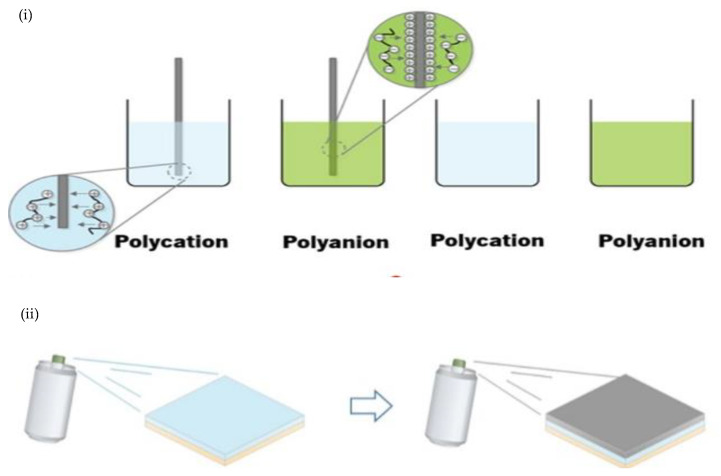
(**i**) Dipping and (**ii**) spray coating method [16].

In industry, coating is usually applied in edible active packaging systems, where the system must preserve the quality of products and extend the lifetime of products [104,105]. According to Muxika et al. [96], chitosan edible coating is used and tested on vegetables and fruits, and there are two ways to apply the coating: dipping and spraying. Dipping is introduced in the food product through acidic solution forming and spraying, introducing the method of pulverizing the film-forming solution [106] (Figure 6). There is difference between them, as they are affected by coating properties or thickness. The formation of polymeric coatings via spraying systems is influenced by factors such as drying time, temperature, and technique, due to the protonation of chitosan amine groups in acidic environments, which gives chitosan caustic properties that eventually decreases certain sensory qualities [107]. However, even dipping and spraying have their own advantages in controlling the quality of food and bacteria growth, but there are still drawbacks present, and these must be considered before implementing dipping and spray coating [96].

### 4.3. Freeze Casting and Drying

**Figure 7:**
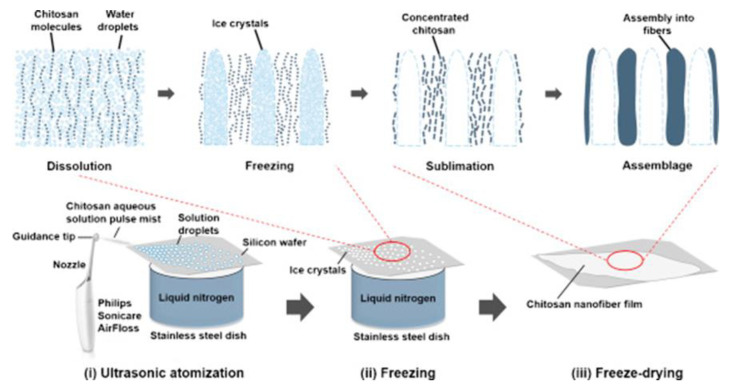
The processes involved in the ultrasonic atomization–freeze casting of chitosan nanofibers [110].

Freeze casting, also known as ice templating, is one of the most common methods that is used in tissue engineering applications in order to obtain chitosan scaffolds. The structure obtained from this technique gives a proper environment for the attachment of cells, growth, and the final form of new production tissue [108]. In addition, this method was created to manipulate the degree of porosity, pore size, pore shape, and pore orientation to modify the pore structure of porous materials [109]. According to Muxika et al. [96], the freeze casting process involves dissolution in a small quantity of acidic aqueous solution followed by freezing the solution in a copper or stainless-steel mold which cools down the solution in a very short time and results in the formation of two distinct phases: the frozen solvent and polymer phase. Wang and Wakisaka [110] used this method in combination with ultrasonic atomization in the production of uniformly oriented chitosan nanofibers (Figure 7). The good mixing of chitosan powder, formic acid, acetic acid, and/or l-lactic acid in distilled water followed by ultrasonic atomization–freeze casting and drying resulted in excellent fiber formability, as well as the minimization of volatile organic solvent use, which made the obtained chitosan nanofibers safe, environmentally friendly and compatible. Because it is a flexible process for manufacturing porous materials, that has attracted a lot of interest in recent years [111,112,113].

### 4.4. Layer-by-Layer

**Figure 8:**
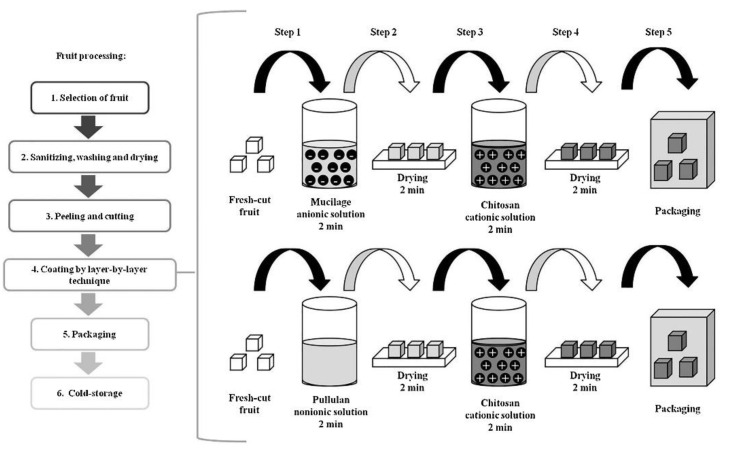
Layer-by-layer technique used in the production of edible coatings based on chitosan, pullulan, linseed, nopal cactus, and aloe mucilage [117].

The layer-by-layer (LbL) deposition technique, which involves building up successive layers of oppositely charged species, has been extensively utilized for producing multilayer, thin films. The electrostatic force of attraction, hydrogen bonding, and affinity between synthetic polymers, proteins, polysaccharides, and other molecules are used in the layer-by-layer technique. According to Costa and Mano [114], because of its cationic character, chitosan has been utilized to create LbL-based films and coatings. Figure 8 shows general processes involved in the LbL method. In this method, substrate is immersed in the chitosan solution, resulting in the formation of a very thin layer on the surface. Numerous properties of the multifunctional chitosan-based films produced can be controlled, namely the thickness difference, permeability to gases and glucose, film strength, as well as film flammability. The characteristics of these films are determined by a few factors such as pH, type of chemical, and ionic cross-linking during deposition, which may influence mechanical film performance [115,116].

### 4.5. Extrusion

**Figure 9:**
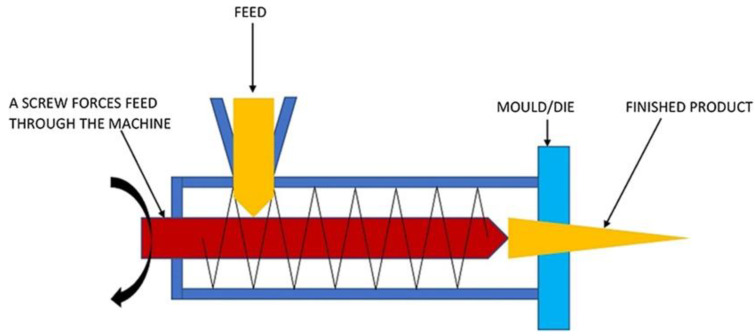
Extrusion method of chitosan film production [123].

Biodegradable, chitosan-based packaging has also been extensively produced via extrusion due to it having a higher productivity and requiring less space. One of the most common extrusion methods is melt extrusion, which produces a film with excellent mechanical characteristics and thermal stability (Figure 9). Basically, this method can produce a final product according to a specific formulation and composition based on requirements. The process involved in this method is simple and involves a continuous flow, where the materials blend in the mixer, followed by an extruder to produce a pellet. The pellets are then used in the production of a film through the twin-screw extruder, usually for medical [118,119], 3D-printing [120], and packaging applications [121,122].

## 5. Mechanical Properties of Chitosan-Based Green Composites

Over the past decade, significant efforts have been made in the development of green composites by utilizing chitosan. This advancement opened the path for future natural-fiber composites (NFCs) with improved mechanical characteristics to be developed by engineers and researchers. Natural fibers such as plant fibers offer a number of benefits, including low weight, cheap cost, and biodegradability. The mechanical characteristics of chitosan-based green composites are important to allow them to be used to their full potential in specific applications. Table 8 lists the mechanical properties of chitosan-based green composites from varying source of fibers.

## 6. Thermal Properties of Chitosan-Based Green Composites

The effect of filler form on composite thermal stability was investigated using a thermogravimetric analyzer (Jupiter STA 449F3, Netzsch). With an initial sample weight of approximately 5 mg, measurements were taken in a nitrogen atmosphere (flow rate 20 cm^3^ min^−1^) at a heating rate of 10 K min^−1^ over a temperature range of 30–1100 °C. Previous research by Grząbka-Zasadzińska et al. [91] used the solvent casting technique to make chitosan/nanocrystalline cellulose composites. To begin, chitosan was dissolved in CH_3_COOH at a concentration of 2% (*v*/*v*). Next, various amounts of nanocrystalline celluloses were applied to chitosan to produce mixtures of CNC mass/mass ratios of 1, 3, and 5% (in comparison to the dry mass of chitosan). Composites of 5% cellulose I and cellulose II were also made as a reference. All of the mixtures were ultrasonically homogenized for 20 min, then added to Petri dishes and dried for 12 h at 35 °C.

**Figure 10:**
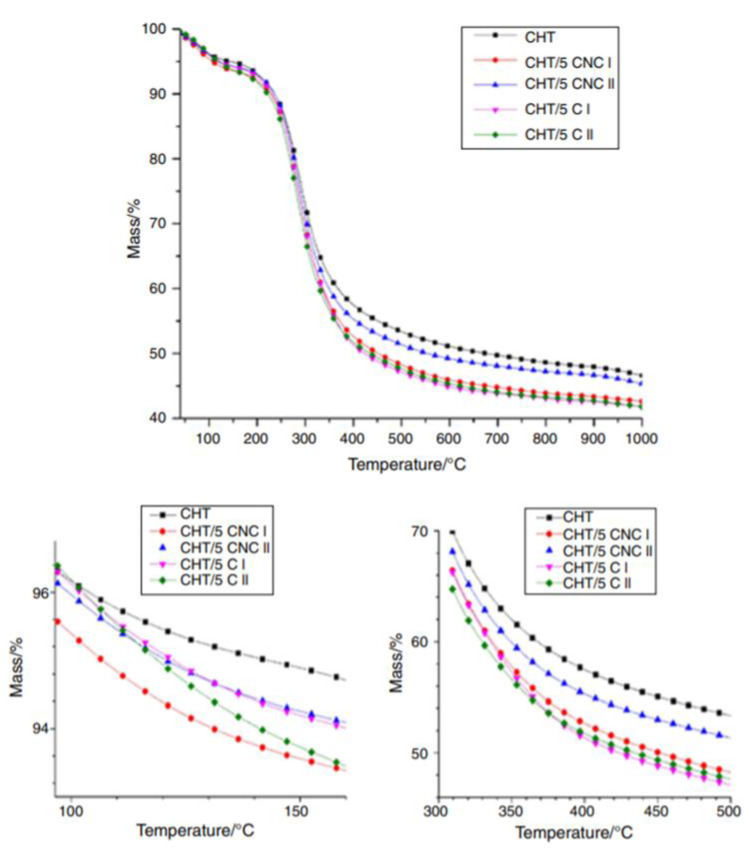
Thermogravimetric curves for chitosan and its composites [91].

The samples were named using the following convention: CHT stands for chitosan, the number represents the percentage of filler added, and C I, C II, CNC I, or CNC II represent the filler form. CHT/5 CNC II, for example, denotes chitosan containing 5% nanocrystalline cellulose II. Thermogravimetric (TG) curves of chitosan and its composites with micro- and nanometric celluloses are given in Figure 10.

The TG findings indicate that the sample containing CNC II has greater thermal stability than the film containing CNC I. However, CNC II composites proved to be more thermally stable than C II composites. A similar trend was observed in composites dependent on (nano)cellulose I, but only when high mass loss was taken into account. In terms of thermal stability, it appears that not only polymorphic variation but also filler size is essential.

## 7. Chitosan-Blend Composites

Despite several benefits of chitosan such as biodegradability, lack of toxicity, and abundance in nature, chitosan-based materials have poor water barrier capabilities owing to their hydrophilic nature, which mainly impacts their mechanical, gas permeability, and thermal properties. Thus, one method for reducing the hydrophilic nature of chitosan is to blend biopolymers to make composites. Blending chitosan with other polysaccharides was found to generate improved barrier, mechanical characteristics, and aesthetic composite properties [127]. This section provides an overview of the research on chitosan-blend composites divided by property characterization, namely mechanical and thermal properties. Table 9 summarizes some of the mechanical properties of chitosan-blend composites for many applications, including packaging and medical applications.

### 7.1. Chitosan-Blend Composites

As reported by Rajan et al. [126], the addition of a high amount of chitosan lowers the crystallinity of Poly(hydroxybutyrate) (PHB) composites, thus decreasing its thermal stability. Similarly, the tensile and impact strengths of composites decrease with the addition of chitosan. On the other hand, some essential oils (such as cinnamon and ginger) can enhance the characteristics of bio-based films of chitosan–carboxymethyl cellulose [128]. These green materials could be used to improve food safety and quality by preserving it.

Thou et al. [129] also pointed out that the mechanical and surface properties of chitosan can be improved by blending it with some carbonaceous materials such as cellulose and multiwall carbon nanotubes [130]. The study also showed that the composites experienced a significant improvement in thermal stability by delaying the degradation time. Cobos et al. [131] synthesized chitosan/graphene (CS/GS) nanocomposites and evaluated their thermal and mechanical properties. CS/GS nanocomposites showed an enhancement in mechanical and thermal properties when compared with native chitosan. This might be due to strong interaction between both polymers through covalent/non-covalent functionalization [136,137,138]. Furthermore, a high weight percent of chitosan matrix dissolved in acetic acid, on the other hand, was able to contribute to the integration of a large quantity of nanofiller reinforcement [139]. Delavar and Shojaei [132] reported that nanodiamond/chitosan composites (ND/CS) possessed good mechanical properties (20% better than unmodified materials) through the dispersion of nanodiamond in chitosan matrix and strong intermolecular interactions between them. Therefore, chitosan-blend composites could be an option to be used industrial applications.

### 7.2. Thermal Properties of Chitosan-Blend Composite

**Figure 11:**
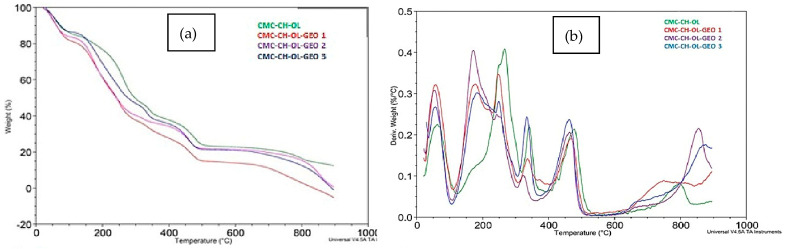
(**a**) TGA for chitosan–essential oil for the first event and (**b**) TGA for chitosan–essential oil and cinnamon essential oil for the second event [129].

The effect of cinnamon and ginger essential oils (EO) on thermal properties of chitosan–carboxymethyl cellulose films emulsified with oleic acid was studied by Noshirvani et al. [129]. Various cinnamon and ginger EO levels were used in the production of biobased films. From the thermogravimetric results, it was found that the combination of oleic acid and EO causes a reduction in thermal stability; the thermal stability decreased as the volume of EO increased, as shown in Figure 11. This is due to the fact that the polymer network’s composition changes, resulting in the formation of a discontinuous structure. This leads to a reduction in the density of the framework and an expansion of free volume locations. Given the presence of EOs, this result may be ascribed to a potential link between cinnamaldehyde and chitosan, which was destroyed as a consequence of the second event.

The incorporation of chitosan into PVA films seems to be a viable approach for obtaining antibacterial and biodegradable food packaging [140]. Bonilla et al. [135] found that the thermal stability was increased in the chitosan blend films. From the TGA analysis, the addition of chitosan increased the T_max_ of the blends, indicating that the PVA films were more thermally stable as a consequence of the chitosan addition. This is due to the fact that the thermal behavior of chitosan, and particularly its glass transition temperature (T_g_), is higher. Furthermore, polymer interactions result in an increase in effective mean molecular weight, and therefore, T_g_ values as a consequence of hydrogen bond formation. The increase in T_g_ values in the blends also indicates that the two macromolecules are miscible. The excellent miscibility of the polymers has an impact on the PVA crystallization process. This decrease in crystallization or melting temperature is indicated the compatibility of the chitosan blend.

## 8. Chitosan Hybrid Composite

This section reviews and discusses the chitosan hybrid composite. The sources of hybrid materials could be synthetic fiber, natural fiber, clay, mineral, polymer, and nanomaterials.

### 8.1. Mechanical Properties of Chitosan Hybrid Composites

Table 10 shows the mechanical properties of chitosan hybrid composites. Arumugam et al. [141] investigated the hybridization of a glass fiber (GF)/sisal fiber (SF)/chitosan (CTS) hybrid composite for orthopedic bone fracture plate applications in future. The composites possessed high mechanical properties due to a unique sandwich structure. It exhibited the bending strength of 343 MPa, ultimate tensile strength of 146 MPa, and compressive strength of 380 MPa with a higher Young’s modulus in the bending tests (21.56 GPa) compared to the tensile (6646 MPa) and compressive modulus (2046 MPa). On the contrary, green composites of chitosan and calcium phosphate were hybridized and resulted in the significant reduction in strength and modulus [141]. The optimal composition (in terms of initial strength and degradation behavior) weight to volume ratio of chitosan/calcium phosphate was 10 wt/v%. In addition, a hybrid composite of chitosan and clay was successfully synthesized via electrostatic interaction between positively charged chitosan and negatively charged clay [142]. The hybridization of both materials improved the mechanical strength and anti-fatigue properties of the composites. Guo et al. [143] incorporated nanostructured hydroxyapatite with chitosan (HA–CS) and investigated their mechanical properties. They pointed out that the hybrid composites have great potential for bone tissue engineering due to excellent biocompatibility and mechanical properties.

### 8.2. Thermal Properties of Chitosan Hybrid Composites

An experiment that was performed by Yeh et al. [154] showed the thermal properties of chitosan hybrid materials with different weights of tetraethoxysilane/vinyltriethoxysilane (VTES/ TEOS). Table 11 indicates that the hybrid materials all possessed better thermostability and thermal decomposition. They improved with an increase in the amount of VTES and TEOS as reticular inorganic SiO_2_ was formed. The hybrid material that was made of TEOS was higher in thermostability than that of VTES because it is difficult for SiO_2_ to take shape in poorly soluble VTES.

**Figure 12:**
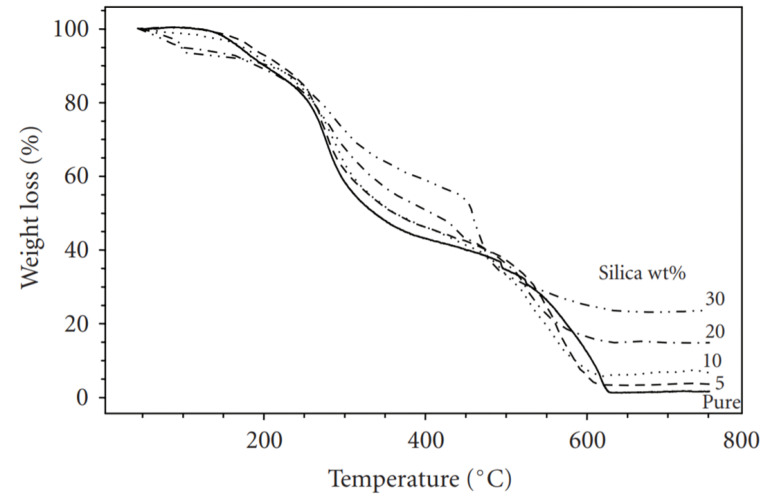
Thermogravimetric curves for chitosan-Si nanocomposites [156].

Because of the decomposition of low-molecular-weight species, chitosan loses weight more slowly between 160 °C and 270 °C. Thermal decomposition is more pronounced between 170 °C and 450 °C, owing to the complex dehydration of the saccharide rings, depolymerization, and decomposition of the polymer’s acetylated and deacetylated units [155]. Yeh, Chen, and Huang [154] studied the effect of silica with chitosan and analyzed the thermogravimetric of nanocomposites, and it was under synthetic air in the temperature range of 50–750 °C. Figure 12 shows the result of TGA.

The initial weight loss observed between 100 and 160 °C tends to be caused by the loss of absorbed water on the surface of chitosan as well as a byproduct of subsequent condensation of the Si–OH groups. Because of the decomposition of low-molecular-weight species, chitosan loses weight more slowly between 160 °C and 270 °C. Thermal decomposition is more pronounced between 170 °C and 450 °C, owing to the complex dehydration of saccharide rings, depolymerization, and the decomposition of the polymer’s acetylated and deacetylated units.

The incorporation of a silica network and its contact with the polymer increases the hybrids’ thermal tolerance and, as a result, the thermal decomposition temperature. The amount of silica material incorporated in the hybrids correlated to the amount of residue retained at 750 °C, suggesting that the sol–gel reaction was active [157].

Thermal properties of potato starch mixed with chitosan films were found to be higher with the addition of citric acid (CA) [147]. The existence of a crosslinking effect with CA addition added to the thermal stability of this film, which may further improve the interactions between molecules. When CA was included in the films, the maximum temperature corresponding to each step in the TGA analysis appeared to emerge at higher temperatures, which may indicate greater intermolecular interactions among the components. On the other hand, Yadav, Behera, Chang, and Chiu [148] studied the thermal properties of cellulose nanocrystal/chitosan (CNC/CS) composite films by varying the CS loading of 2, 4, 6, and 8 wt.%. The TGA results showed that the incorporation of CS at different loadings showed almost the same thermal stability. Due to interactions between the CNC and the CS matrix, the thermal stability of CNC-reinforced CS composite films improved marginally after CNC insertion.

Thakhiew, Devahastin, and Soponronnarit [149] developed a blended film of chitosan and galangal rhizome extract at different drying methods, i.e., hot-air drying (HAD) and low-pressure, superheated steam drying (LPSSD), and different loadings of galangal rhizome extract, i.e., 0%, 0.6%, 0.9%, 1.2%, and 1.5%. They found that the usage of a higher temperature in the LPSSD may have resulted in more widespread thermal cross-linkage compared to the HAD method. In the case of the incorporation of galangal extract, a higher number of chemical cross-linkage interactions between the galangal extract and chitosan was observed. These phenomena were due to the electrostatic interactions and hydrogen bonding that may have caused conformational changes when the galangal extract was integrated into the chitosan matrix. From the DMA analysis, the storage modulus, loss modulus, and tan δ of the chitosan films across a temperature range of −120 to 230 °C were obtained, which show evidence of cross-linkage interactions.

Deepmala, Jain, Singh, and Chauhan [150] manufactured chitosan-coated, human-hair-reinforced, phytagel-modified, soy-protein-based composite for packaging and coating applications. Various wt.% of human hair were used with coated and non-coated chitosan. The experimental data prove that the tensile stress was enhanced to 24.54 MPa with the application of chitosan coating, due to the fact that the maximum surface cracks and voids were filled with the chitosan solution and the stress concentration was decreased. In the view of thermal properties, chitosan coating was found to improve the storage modulus and the glass transition temperature; 2 wt.% human hair had the highest value of storage modulus but the lowest value of tan δ. The stiffness of the manufactured samples was enhanced as a result of the chitosan coating application on the final composites, as the non-coated surface contained many cracks, while chitosan coating covered all gaps and surface cracks of the composite’s surface. Because the energy dissipation process was ineffective in this case, composites started to behave like brittle materials, thus increasing stiffness and storage modulus.

**Figure 13:**
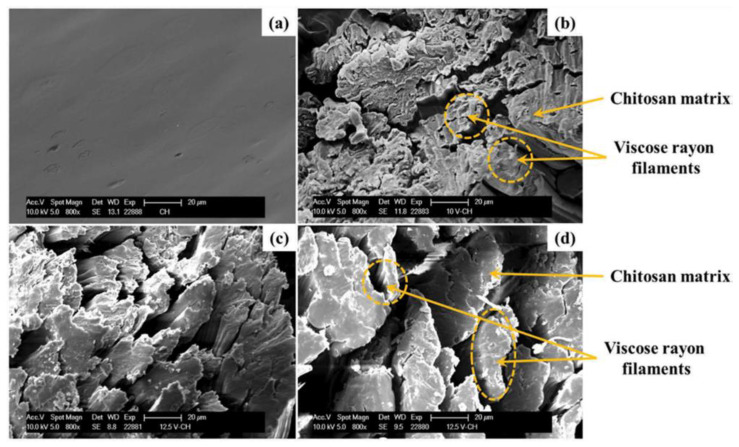
The micrograph images of (**a**) chitosan film, chitosan–viscose rayon biocomposite with different loadings; (**b**) 12 wt.%, (**c**) 20 wt.%, and (**d**) 25 wt.%.

**Figure 14:**
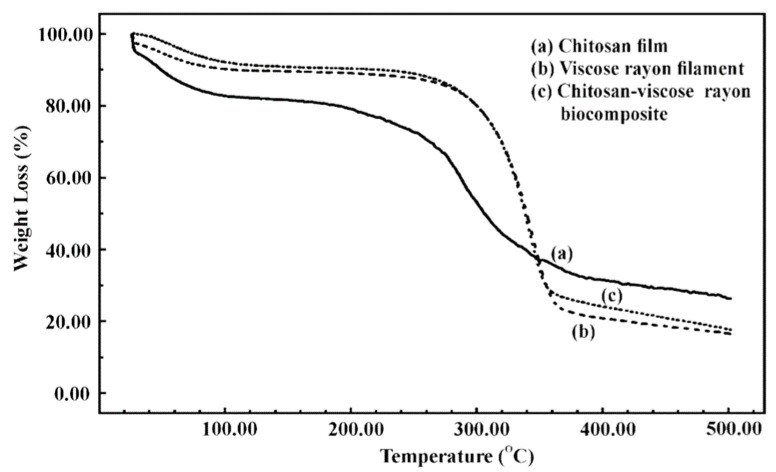
The TG curves of (**a**) chitosan film, (**b**) viscose rayon filament, and (**c**) chitosan–viscose rayon biocomposite.

Gorade, Chaudhary, Parmaj, and Kale [151] prepared a composite with viscose rayon filaments and reinforcement with chitosan to produce a chitosan–viscose rayon biocomposite. Various weights of viscose rayon filament (15, 20 and 25 wt.%) were examined in the view of microstructure and thermal properties. As the weights of viscose rayon filament increased, some voids were observed due to the fact that the chitosan solution did not penetrate completely during biocomposite production, as shown in Figure 13. However, in terms of thermal stability, the result of including viscose rayon filament into the chitosan polymeric matrix increased the biocomposite’s thermal stability owing to viscose rayon filament’s greater thermal stability, as shown in Figure 14. The sample decomposition generally involved breaking down of glycosidic units into smaller pieces, followed by the production of volatile gases. In addition, the glycosyl unit was completely decomposed and depolymerized, resulting in the production of char [158].

Prabhakar and Song [152] examined the composite prepared by hybridizing corn starch, chitosan, and flax fabric to produce effective flame-retardant, eco-biodegradable composites for industrial applications. They concluded that the decomposition at higher temperatures from the TGA analysis shows that corn starch has a beneficial effect on the composites’ thermal stability due to the multihydroxyl groups in corn starch, which may create additional molecular hydrogen bonds. In terms of flammability, the flame retardancy improved substantially as the amount of corn starch increased. In addition, the thick char produced by the carbonaceous ingredient corn starch was thought to be responsible for the composites’ flame-retardant characteristics.

**Figure 15:**
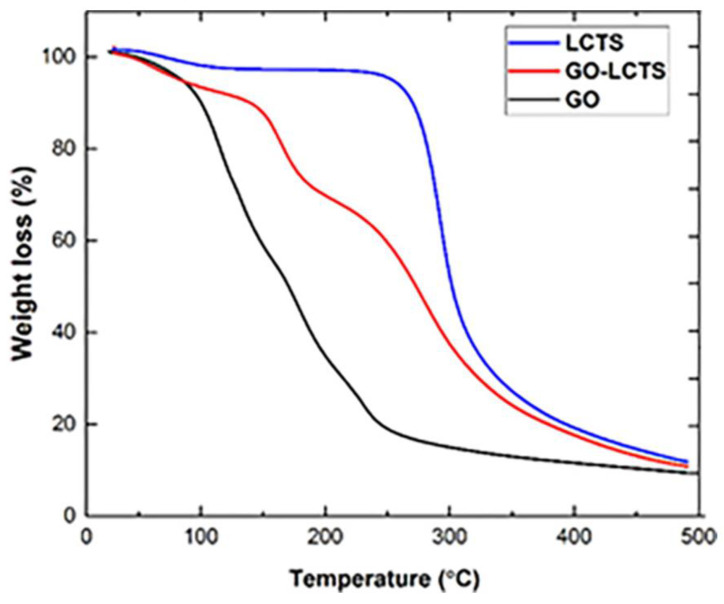
The TGA curves of graphene oxide (GO), chitosan (LCTS), and GO-LCTS composite [159].

Sabzevari et al. [159] found that the graphene oxide (GO) chitosan composite has greater thermal stability over GO, as shown by its stability upon heating to the upper-temperature limit of 500 °C (Figure 15). In this study, low-molecular-weight chitosan was cross-linked with GO to produced GO–chitosan composite. The higher thermal stability of the GO–chitosan composite was due to higher crystallinity observed from the X-ray diffraction (XRD) and scanning electron microscope (SEM) analyses. The chitosan chains were well introduced and firmly bound to the oxygen functional groups of GO, while preserving the stacked structure of GO sheets in the material, which indicated excellent cross-linking occurred.

Verma, Singh, Singh, and Jain [153] conducted an experiment on hybrid composites reinforced with soy protein and sisal fiber by varying sisal fiber weight percentages of 0, 3, 4, 5, 6, 7, and 10. In this study, chitosan was used as a coating material, where the composites were coated with chitosan by immersing the samples in chitosan solution. They reported that the inclusion of sisal fiber at a higher weight percentage and chitosan coating to the thermal tests resulted in an improvement in thermal stability. The DMA analysis also demonstrated that the storage modulus and glass transition temperature for different compositions were greater for chitosan-coated specimens than for non-coated specimens, with the maximum values observed at 5 wt. percent sisal fiber composites in both instances.

## 9. Application of Chitosan-Based Green Composites

Chitosan has many usages or applications in industries, for example, biomedicine, cosmetology, papermaking, wastewater treatment, agriculture, or pharmaceutical applications, and others. The applications are further explained in the next section, which consists of an explanation for biomedical applications and the specific usage of chitosan in the industry. Chitosan has many benefits towards biomedical applications such as biocompatibility and control biodegradability, which lead to the degradation of products. In addition, it is not harmful and does not produce any dangerous reactions [96]. Besides that, chitosan can be modified by blending it with other polymeric materials such as cellulose because it has modifiable functional groups; thus, the stability of blends enhances. Some of the potential applications of chitosan–cellulose blends are tabulated in Table 12. It can be summarized that the stability of chitosan-based materials can be improved by blending them with other compatible biopolymers that can be commercially utilized.

### 9.1. Drug Delivery

**Figure 16:**
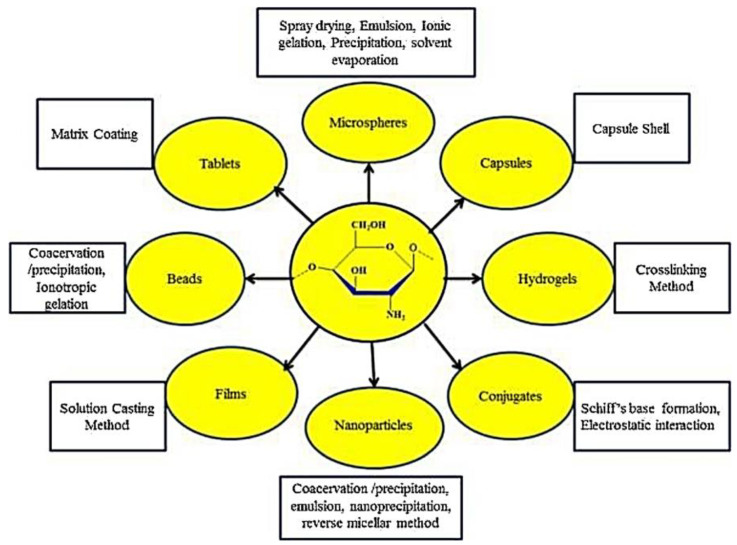
Drug delivery systems based on chitosan and various techniques for their manufacture [181].

Nowadays, controlled drug delivery provides many advantages to humans, e.g., it can enhance efficacy and reduce or eliminate unwanted side effects and also the level of drugs. Chitosan has some special properties that make it ideal to be used for drug delivery functions. Furthermore, a chitosan nanoparticle system that is conjugated by anti-bradykinin B2 can enhance the inhibition of HIV replication. Chitosan used in the drug industry is solely used to reduce side effects from cancer treatment such as cardiotoxicity. The drug is confined with chitosan nanoparticles. These nanoparticles of chitosan can enhance the absorption of a chemical known as doxorubicin in the small intestine. The type of nanoparticles of chitosan that is commonly used in this specific industry is chitosan tripolyphosphate (TPP) nanoparticles [178]. Chitosan nanoparticles also can improve the tea polyphenols stabilities and prevent their reactions of oxidation or degradation in the gastrointestinal tract. Hence, chitosan that synthesized from polyethylene glycol is probably suitable for use as a drug controlled release carrier [96]. According to Bernkop-Schnürch and Dünnhaupt [179] and Ahsan et al. [180], the chemical stability, particle sizes, toxicity, release kinetic profiles, and type of delivery system are important elements that must be considered when it comes to processing chitosan for this purpose. Chitosan can be used in various forms depending on the function and applications of the carrier. Figure 16 shows that chitosan can be employed in a variety of ways, depending on the carrier’s function and uses.

### 9.2. Wound Dressing

Chitosan is a natural antibacterial polymer with features that make it excellent for wound dressing, such as being cheap to make, stable for long-term use, biodegradable, non-toxic, and having a biocidal impact on a wide range of pathogens. Besides, chitosan is a suitable material or substance for wound dressing, mainly for the prevention of wound infection. It is suitable or compatible due to inherent antibacterial activity and other benefits such as analgesic effects and hemostatic activity. Plus, chitosan can function as a mechanical barrier on blood that causes immediate clotting. Chitosan efficacy was tested and carried out in vitro toxicity evaluation on 3T3 cells, and the result showed that it is an agent suitable for the treatment of internal and external bleeding. Additionally, chitosan-based products vary from typical dressings such as gauze or cotton wool in that chitosan actively participates in wound healing processes [182]. Chitosan-based wound dressing materials have been formed into a number of shapes, including films, sponges, hydrogels, particles, and fibers, due to its ease of processing, as shown in Table 13. Matica et al. [183] comprehensively discussed chitosan as a wound dressing in medical sectors.

### 9.3. Food Packaging

In the food industry, chitosan is considered as a bioactive polymer that can be used for food packaging manufacture based on its antioxidant, antimicrobial, mechanical, and barrier properties. The use of chitosan as a material for packaging can reduce the risk on human health and also environmental problems. In fact, the mechanical and barrier properties of pure chitosan films are suitable for food and active packaging. Chitosan has also been made a reference polymer in order to manufacture active packaging with the improvement or capability to prevent the growth of microorganisms and upgrade the safety of food [178]. It is feasible to create food packaging items that are safe against a broad range of modifying and pathogenic microbes by mixing chitosan with other natural antimicrobial agents [178,207,208].

**Figure 17:**
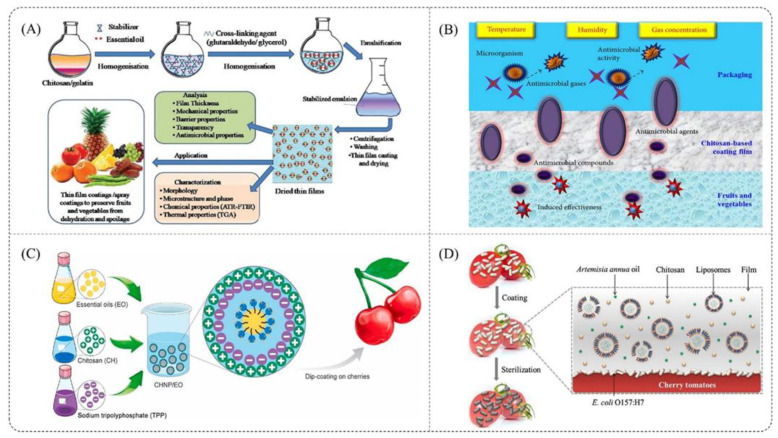
(**A**) Development of chitosan/gelatin-based polymeric films with inclusion of citrus essential oils [209]; (**B**) preservation mechanism of chitosan-based coating to maintain quality of vegetables and fruits [210]; (**C**) multifunctional coating composed of Eryngium campestre essential oil encapsulated in nano-chitosan to prolong the shelf-life of fresh cherry [211]; (**D**) edible film’s antimicrobial activity against E. coli O157:H7 on cherry tomatoes [212]. Reproduced from Zhang et al. [213].

Chitosan also can be used as a thin edible film/coating with food several methods such as by dipping, spraying, and pulverizing the film forming solution with an aerosol spray coating [214,215,216], as shown in Figure 17. An edible coating is a thin layer generated as a coating on a food product that is applied in liquid form, while chitosan film is a prefabricated thin coating that may be applied on or between food components once produced. Because chitosan regulates gas exchange, inhibits the respiration rate, is fungistatic, is capable of eliciting host defense responses, and lowers the rate of ethylene generation compared to the control fruit throughout the storage period, it has the potential to extend the shelf life of perishable fruits and vegetables as a post-harvest treatment [106,217].

Researchers found that chitosan coating significantly preserves the qualitative qualities of sliced mango fruit and extends its shelf life by reducing water loss and sensory degradation, boosting soluble solid content, titratable acidity, vitamin C, and preventing microbe development [218,219,220,221]. Table 14 shows studies made with chitosan films and chitosan coatings in food products.

### 9.4. Dermatology and Skin Care

In cosmetic applications, chitosan is a natural cationic polymer which turns viscous when being neutralized with acid and works as a cationic humectant in cosmetics and topical formulations. It is used in the manufacture of creams, lotions, and other cosmetic preparations. Additionally, chitosan is known for its application as a film-forming and hydrating agent. Besides, chitosan also has benefits as sun protection, as the emulsions of sun protection that were mixed with chitosan have good effects on water resistance, which improve the safety of skin [230]. By changing the keratin structure, chitosan is extensively employed as a skin permeability enhancer in drug delivery systems, and it is absorbed to the negative charges of the skin surface [231]. Chitosan also improves the water content of the stratum corneum and increases the fluidity of the cell membrane, aided by its hydrophilic hydroxyl groups, which enable chitosan to interact with water molecules [232]. Chitosan adheres to the skin due to its positive charges and relatively high molecular weight, allowing it to be used as a percutaneous drug delivery vehicle. In recent years, there has been increased interest in using chitosan in the creation of nanoparticles as a carrier for active ingredients in cosmetics and medicine delivery to the skin. Chitosan-based nanoparticles have been used to treat local problems including skin malignant melanoma and infection [233].

### 9.5. Cosmetics for Oral Care Products

As oral care products affect human health, the cosmetics industry tends to use and focus on natural compounds such as chitin, chitosan, and their derivatives. Since chitosan has a lower molecular weight, it shows inhibition on the oral adsorption of streptococci and is proposed as a potential anticavity agent. Chitosan also able to interfere with microorganisms’ adherence and other factors [230]. Achmad et al. [234] evaluated the efficacy of chitosan against dental plaque development in actual formulations. In the study, chitosan was incorporated into toothpastes, rinses, and other vehicles. Chitosan, which is used in toothpaste and mouthwashes to prevent biofilm development in the mouth owing to the presence of *S. mutans*, has been shown to reduce *S. mutans* colonies. Chitosan has a broad antibacterial spectrum; thus, its efficacy against various bacterial strains associated with dental caries has been studied by a number of researchers [235,236,237].

## 10. Challenges and Opportunities

Chitosan captures a special position as a natural source in the composite industry due to its appealing characteristics such as antibacterial and film-forming properties. Currently, many products and applications are utilizing chitosan, primarily in the food and medical lines. Extensive research and manufacturing efforts have supported the development of chitosan-based products, which has been aided by rising customer demand for natural and safer additives with useful qualities, as well as rising environmental concerns [238,239]. However, there are few challenges that need to be focused on to widen the application of chitosan-based composites. The methods or techniques involved in chitosan-based products are mostly for small-scale production. To move closer to industrial production, these current technologies may be improved or integrated with other beneficial technology in order to produce mass numbers of chitosan-based products with the necessary characteristics for various applications. Due to some chitosan limitation properties such as low thermal stability to fulfil specific needs, more chitosan derivatives must be researched, and the degradation and environmental impact of chitosan-based products must be studied. Furthermore, more research is needed, especially in terms of toxicity and antibacterial properties, to improve chitosan-based products for food packaging before they may be used commercially. Improvements in thermal and strength characteristics, as well as the ability to bind contaminants, are also required.

## 11. Conclusions

Nature provides a variety of biomaterials that can be obtained easily from animals and plants. Chitosan is one natural fiber with promising characteristics as a composite material. As a biomaterial form, chitosan can be made into a few forms such as 3D (hydrogels and sponges) and 2D (films and porous membranes); each of them have their own set of industrial applications. On the other hand, chitosan has relatively poor mechanical, thermal, and barrier properties. For example, chitosan experienced weight reduction (50%) at 220–320 °C and a further 40% weight loss at the temperature of 470 °C. With the combination of two or more polymers, biomaterials can significantly increase the properties of composites. Therefore, chitosan-based composites are extensively investigated and explored. There are various methods available in the literature according to applications for the development of chitosan-based composites, including solution and solvent casting, dipping and spray coating, freeze casting and drying, layer-by-layer processes, and extrusion. The mechanical properties and thermal properties of different types of composites have been discussed from different resources. It was reported that the developed chitosan-based green composites, chitosan-blend composites, and chitosan hybrid composites showed thermal stability improvement due to the highly crystalline structure of the composites observed by XRD and SEM analysis. Not only that, but their mechanical properties also enhanced with the increase in tensile strength. As chitosan green composites offer many advantages, they have a wide range of applications and potential in the biomedicine, cosmetology, papermaking, wastewater treatment, agriculture, and pharmaceutical industries. In future, the use of chitosan-based composites in industrial applications may be able to completely replace synthetic fibers, thus reducing environmental pollution. In-depth research is still needed in terms of investigating the environmentally friendly chemical treatment used, as well as the toxicity and antibacterial properties of materials, so that composite materials reinforced with natural fibers can perform better in the future.

## Figures and Tables

**Table 1:** Examples of natural fibers [49].

Natural Fibers	Example
Mineral	Asbestos
	Fibrous brucite
	Wollastonite
Plant	Bast Flax Hemp Kenaf Jute
	Leaf Sisal Banana
	Fruit Cotton Coir
	Grass Bamboo Indiangrass Switch grass
	Straw Corn Rice
	Wood pulp
Animal	Silk
	Wool
	Feathers

**Table 2:** Production amount of natural fiber produced [56].

Fiber	Producer	Production Amount (×10^3^ ton)
Abaca	Philippines (85%), Ecuador	70
Alpaca	Peru, Bolivia, Chile	7
Angora wool	China, Argentina, Chile, Czech Republic, Hungary, France	3
Bagasse	Brazil, China, India, Thailand, Australia, USA	75,000
Bamboo	China, Japan, India, Chile, Ecuador, Indonesia, Myanmar, Nigeria, Sri Lanka, Philippines, Pakistan	30,000
Camel hair	China, Mongolia, Afghanistan, Iran	2
Cashmere wool	China, Mongolia, Australia, India, Iran, Pakistan, New Zealand, Turkey, USA	20
Coir	India, Sri Lanka, Thailand, Vietnam, Philippines, Indonesia, Brazil	1200
Cotton	China, Brazil, India, Pakistan, USA, Uzbekistan, Turkey	25,000
Flax	France, Belgium, Netherland, Poland, Russian Federation, China	830
Hemp	China (80%), Chile, France, Germany, UK	214
Jute	India (60%), Bangladesh, Myanmar, Nepal	3450
Kapok	Philippine, Malaysia, China, South America, Indonesia, Thailand	101
Kenaf	India (45%), China, Malaysia, USA, Mexico, Thailand, Vietnam	970
Mohair wool	South Africa, USA	5
Ramie	China, Brazil, Lao PDR, Philippines, India	280
Silk	China (70%), Brazil, Bulgaria, Egypt, Madagascar, India, Thailand, Vietnam, Uzbekistan, Turkmenistan	150
Sisal	Brazil (40%), Kenya, Tanzania, China, Cuba, Haiti, Madagascar, Mexico, Sri Lanka, India	378
Wool	Australia, Argentina, China, Iran, New Zealand, Russia, UK, Uruguay	2100

**Table 3:** Chemical composition of lignocellulosic fiber [18].

Type of Fiber	Cellulose	Hemi Cellulose	Lignin	Pectin	Wax	Ash	Moisture	Others
Abaca	56–64	25–29	11–14	-	-	-	-	-
Jute	64.4	12	0.2	11.8	0.5	0.5–2.1	10	-
Sisal	65.8	12	0.8	9.9	1.2	0.3	10	-
Kenaf	44.4	-	20.1	-	-	4.6	-	-
Coconut	37–43	24–28	26–28	-	-	-	-	7
Bamboo	78.83	-	10.15	-	-	-	-	-

**Table 4:** Physical and mechanical properties of lignocellulosic fibers [18].

Type of Fiber	Diameter (μm)	Density (g/cm^3^)	Tensile Strength (MPa)	Young’s Modulus (GPa)
Abaca	250–300	1.5	717	18.6
Jute	250–2500	1.3–1.49	393–800	13–26.5
Sisal	205–230	1.41	350–370	12.8
Kenaf	83.5	1.2	282.60	7.13
Coconut	396.98	1.2	140–225	3–5
Bamboo	-	1.2–1.5	500–575	27–40

**Table 5:** Advantages and disadvantages of biomaterials for chitosan [75].

Type	Descriptions	Advantages	Disadvantages
Hydrogels (3D)	- Physically related (reversible) - Chemically cross-linked (irreversible)	- Soft, flexible, and safe - Soft, flexible, and has stable porous size	- Not stable, low mechanical resistance and hard to control the pore size - Toxic
Sponges (3D)	- Free sanding	- High porosity and soft	- May dry up and low porosity
Films (2D)	- Thin (LB) - Thin (LBL)	- Coat material - Coat material, multiple layer construction	- Difficult for the construction of many layers - Has many steps
Porous Membrane (2D)	- Nano fibers	- High porosity, mimics skin, and extracellular matrix	- Hard for pure chitosan

**Table 6:** Physical properties of chitosan-based polymer.

Type of Chitosan-Based	Physical properties	Explanation	References
Chitosan—tapioca starch edible film	Water vapor permeability Water solubility	Determined gravimetrically Solubilized in distilled water	[83]
Chitosan film—natural antioxidants	Surface color measurement Opacity and transparent Water content, solubility and swelling degree	Measurement of CIE-L*a*b* coordinates Spectrum scan using UV/VIS spectrophotometer	[84]
Chitosan—green tea extract	Film color and opacity Water vapor permeability coefficient and density	Using effects of GTE concentration	[85]

**Table 7:** Mechanical properties of chitosan.

Type	Percentage (%)	Elongation (%)	Tensile Strength (MPa)	Young’s Modulus (GPa)	References
Chitosan (CS)	2.0–10.0 (CS)	-	9.0–16.0	250–380	[87]
Chitosan—antimicrobial	2.0–10.0 (CS)	-	14.0–18.0	150–440	[87]
Chitosan—Spirulina Extract (SE)	2.5–50.0 (SE)	26.13–39.53	21.24–29.65	-	[88]
Chitosan—graphene oxide (GO)	0.0–2.0 (GO)	57.34–72.70	6.99–15.32	-	[89]
Chitosan—glycerol	1.0–3.0 (CS)	9.50–67.93	0.281–12.147	-	[90]

**Table 8:** Mechanical properties of chitosan-based green composites.

Polymers	Fibers	Processing Technique	Mechanical Properties		References
			Tensile Strength	Tensile Modulus	
Chitosan	Cellulose-modified	Ionic liquid treatment	22–80 MPa	236–3316 MPa	[91]
Chitosan	Bamboo charcoal	Blending	25–75 MPa	4600–5400 MPa	[124]
Chitosan	Modified bamboo charcoal	Blending	75–110 MPa	5400–7000 MPa	[124]
Chitosan	Thyme	Dissolution	5.59–12.2 MPa	-	[125]
Chitosan	Clove	Dissolution	6.54–12.2 MPa	-	[125]
Chitosan	Cinnamon	Dissolution	12.2–21.35 MPa	-	[125]
Chitosan	PLA/CS	Solution casting	30.95 MPa	4.10 MPa	[126]
Chitosan	PLA/CS/ENR	Solution casting	10.0 MPa	4.70 MPa	[126]

**Table 9:** Mechanical properties of chitosan-blend composites.

Polymers	Polymers Blend	Processing Technique	Mechanical Properties		References
			Tensile Strength	Tensile Modulus	
Chitosan	Polyhydroxybutyrate	Melting	7.5–11 MPa	1044–2499 MPa	[128]
Chitosan	Deacetylated chitosan	Gel spinning	59.8–117.1 MPa	2.1–4.1 GPa	[128]
Chitosan	CMC-CH-OL	Magnetically stirring	7.0 ± 0.8 MPa	-	[129]
Chitosan	CMC-CH-OL-CEO	Magnetically Stirring	4.8 ± 0.9 MPa	-	[129]
Chitosan	Carbon nanotubes	Magnetically Stirring	-	-	[130]
Chitosan	Cellulose nano whiskers	Solution casting	21.6–31.25 MPa	399.5–535.76 MPa	[131]
Chitosan	Cellulose nano whiskers	Solution casting	21.6–38.25 MPa	399.5–644 MPa	[131]
Chitosan	Glycerol-free	Solution casting	28–44.5 MPa	1.05–1.15 GPa	[132]
Chitosan	Glycerol-plasticized	Solution casting	22.5–33 MPa	0.6–1.0 GPa	[132]
Chitosan	Nano diamond (4.5–1%)	Solution casting	100 ± 2.5 MPa	3314 ± 416 MPa	[133]
Chitosan	Biogenic silver nanoparticles	Ultra sonication	65.04 ± 1.46 MPa	-	[134]
Chitosan	Poly vinyl alcohol (PVA)	Film-forming dispersions and casting	24–43 MPa	-	[135]
Low and high molecular weight (LMw/HMw) chitosan	Glycerol	Solution casting	LMw CS: 31.89–61.82 MPa HMw CS: 23.87–55.83 MPa	-	[106]

**Table 10:** Mechanical properties of chitosan hybrid composites.

Polymers	Fiber	Processing Technique	Mechanical Properties		References
			Tensile Strength	Tensile Modulus	
Chitosan	Sisal fiber reinforced with hybrid polymer sandwich composite	Layer-by-layer	110–146 MPa	5800–6646 MPa	[141]
Chitosan	Calcium phosphate-flexible chitosan	Mixing and heating	45.7 MPa	-	[142]
Chitosan	Clay–chitosan hybrid	Electro-stimulus-responsive	2.25–2.70 MPa	0.2–1.5 MPa	[143]
Chitosan	Bioactive calcium phosphate-flexible chitosan	Mixing and heating	1.6–45.7 MPa	10.2–77.3 MPa	[142]
Chitosan	Hydroxyapatite	Dip-coating and bio inspired mineralization	3.12 MPa	73.67 MPa	[144]
Chitosan	CS fiber porous scaffold	Dip-coating and bio inspired mineralization	0.68 MPa	3.40 MPa	[144]
Chitosan	Trabecular bone	Dip-coating and bio inspired mineralization	-	-	[144]
Chitosan	Sodium montmorillonite and zinc oxide nanoparicles	Polymer intercalation	22.34 MPa ± 1.75	1.750 MPa ± 0.06	[145]
Chitosan	Nano-ZnO nanocomposite	Polymer intercalation	30.49 MPa ± 1.17	2.190 MPa ± 0.02	[145]
Chitosan	Nano-ZnO and organoclay nanocomposite-C4	Polymer intercalation	38.86 MPa ± 1.49	2.410 MPa ± 0.01	[145]
Chitosan	Grape pomace extract	Solvent casting	9.89–13.58 MPa	0.13–0.20 MPa	[146]
Chitosan	Potato starch	Solution blending/casting	9.27–12.5 MPa	-	[147]
Chitosan	Cellulose nanocrystal (CNC)	Solution casting	79.3–104.7 MPa	1607–2068 MPa	[148]
Chitosan	Galangal rhizome extract	Chitosan film forming solution	46.1–67.5 MPa	-	[149]
Chitosan as a coating material	Soy protein isolated and human hair fibers	Hot pressed and compression molding	11.67–24.54 MPa	-	[150]
Chitosan	Viscose rayon filaments	Film molding	105–151 MPa	1.94–2.43 GPa	[151]
Chitosan	Corn starch and flax fabric	Compression molding	17.64–24.03 MPa	0.63–0.66 GPa	[152]
Chitosan as a coating material	Soy protein and sisal fiber	Hand lay-up and solution casting method	11.67–23.70 MPa	-	[153]

**Table 11:** Thermal properties of chitosan and hybrid materials [154].

Weight of VTES/TEOS (g)	Thermal Properties		
	T_d_ (°C)	T_m_ (°C)	Char Yield (%)
0/0	245	303	34.1
0/0.8	249	306	37.2
0.8/0.8	253	308	40.4
0.8/1.6	257	310	43.8
0.8/2.4	260	313	45.6
0.8/3.2	263	315	47.1
1.2/0	247	304	36.3

**Table 12:** Some of the potential applications of chitosan–cellulose/nanocellulose composites.

Potential Applications	References
Adsorbent for the removal of heavy metal ions	[160,161,162]
Adsorbent for the removal of acidic reagents, metals, amino acids, proteins, and other compounds	[163]
Biocomposite films	[164,165]
Biomedical applications	[166]
Coronary artery bypass graft	[167]
Drug delivery	[168]
Electronic	[169]
Food packaging	[170]
Medical material	[171]
Odor treatment	[172]
Self-healing	[173]
Textiles	[174]
Wound dressing	[175,176]
Wound healing (good antibacterial effect)	[177]

**Table 13:** The benefits and drawbacks of major wound dressing types [183].

Type	Advantages	Disadvantages	Refs
Sponges	- high porosity - thermal insulation - sustain a moist environment - absorb wound exudates - enhance tissue regeneration	- mechanically weak - may provoke skin maceration - unsuitable for third-degree burn treatment or wounds with dry eschar	[183,184,185,186,187,188]
Films	- impermeable to bacteria - allow the healing process to be monitored - painless removal	- hard to handle - non-absorbent - adhere to the wound bed and cause exudate accumulation	[189,190,191,192]
Fibers	- non-adherent - high porosity and absorption capacity - mimic the skin’s extracellular matrix	- unsuitable for third-degree, eschar, and dry wounds - if the wound is highly exudative, it needs a secondary dressing	[75,193,194,195,196,197,198]
Membranes	- act as physical barriers - membranes simulate extracellular matrix (ECM) structure - ensure gas exchange, cell proliferation, and nutrient supply	- the materials and solvents used in the production - process may be harmful	[199,200,201]
Hydrogels	- high absorption properties - provide a moist environment at the wound site - water retention - oxygen permeability - ensure the solubility of growth factor/antimicrobial agents	- weak mechanical properties - need a secondary dressing	[202,203,204,205,206]
Hydrocolloids	- non-adherent - high density - painless removal - high absorption properties	- can be cytotoxic - have an unpleasant odor - low mechanical stability - maintain acidic pH at the wound site	[183]

**Table 14:** Applications of chitosan-based films and coatings in different food products.

Chitosan Based	Combination	Food	References
Film	Gelatin/grape seed extract/*Ziziphora clinopodioides* essential oil	Minced trout fillets	[222]
	Chitosan powder/glycerol/NaOH solution	Chilled meat	[115]
	Cassava starch/glycerol/polyethylene glycol	Meat slices	[223]
	Zataria multiflora essential oil/Cinnamomum zeylanicum	Green chili	[224]
	Chitosan powder/glycerol	Chilled meat	[225]
	Chitosan/ Basil Essential Oil	Cooked ham	[226]
	Apricot (Prunus armeniaca) kernel essential oil/glycerol	Spiced beef	[227]
Coatings	Agar/*Artemisia annua* oil	Cherry tomato	[212]
	Apple peel polyphenols (APP)/glycerol	Strawberry	[228]
	Essential oils (EO) of Elettaria Cardamomum/glycerol	Chicken drumsticks	[229]
